# Prescription Drug Dispensing and Patient Costs After Implementation of a No Behavioral Health Cost-Sharing Law

**DOI:** 10.1001/jamahealthforum.2024.0198

**Published:** 2024-03-22

**Authors:** Ezra Golberstein, James M. Campbell, Johanna Catherine Maclean, Samantha J. Harris, Brendan Saloner, Bradley D. Stein

**Affiliations:** 1 Division of Health Policy and Management, University of Minnesota School of Public Health, Minneapolis; 2 Schar School of Policy and Government, George Mason University, Fairfax, Virginia; 3 Department of Health Policy and Management, Johns Hopkins Bloomberg School of Public Health, Baltimore, Maryland; 4 RAND Corporation, Pittsburgh, Pennsylvania; 5 National Bureau of Economic Research, Cambridge, Massachusetts

## Abstract

**Importance:**

On January 1, 2022, New Mexico implemented a No Behavioral Cost-Sharing (NCS) law that eliminated cost-sharing for mental health and substance use disorder (MH/SUD) treatments in plans regulated by the state, potentially reducing a barrier to treatment for MH/SUDs among the commercially insured; however, the outcomes of the law are unknown.

**Objective:**

To assess the association of implementation of the NCS with out-of-pocket spending for prescription for drugs primarily used to treat MH/SUDs and monthly volume of dispensed drugs.

**Design, Settings, and Participants:**

This retrospective cohort study used a difference-in-differences research design to examine trends in outcomes for New Mexico state employees, a population affected by the NCS, compared with federal employees in New Mexico who were unaffected by NCS. Data were collected on prescription drugs for MH/SUDs dispensed per month between January 2021 and June 2022 for New Mexico patients with a New Mexico state employee health plan and New Mexico patients with a federal employee health plan. Data analysis occurred from December 2022 to January 2024.

**Exposure:**

Enrollment in a state employee health plan or federal health plan.

**Main Outcomes and Measures:**

The primary outcomes were mean patient out-of-pocket spending per dispensed MH/SUD prescription and the monthly volume of dispensed MH/SUD prescriptions per 1000 employees. A difference-in-differences estimation approach was used.

**Results:**

The implementation of the NCS law was associated with a mean (SE) $6.37 ($0.30) reduction (corresponding to an 85.6% decrease) in mean out-of-pocket spending per dispensed MH/SUD medication (95% CI, −$7.00 to −$5.75). The association of implementation of NCS with the volume of prescriptions dispensed was not statistically significant.

**Conclusions and Relevance:**

These findings suggest that the implementation of the New Mexico NCS law was successful in lowering out-of-pocket spending on prescription medications for MH/SUDs, but that there was no association of NCS with the volume of medications dispensed in the first 6 months after implementation. A key challenge is to identify policies that protect from high out-of-pocket spending while also promoting access to needed care.

## Key Points

**Question:**

Did out-of-pocket spending and dispensing of prescription drugs change after a state law that prohibited cost-sharing for mental health and substance use disorder treatments was implemented?

**Findings:**

In this cohort study of prescription data from 47 229 individuals that used a difference-in-differences analysis to examine dispensing and cost data for mental health and substance use disorder medications, the New Mexico No Behavioral Cost Sharing law was associated with a significant 85.6% reduction in patient spending per medication. The volume of medications dispensed was unchanged.

**Meaning:**

These findings suggest that eliminating cost-sharing for mental health and substance use disorder treatments can greatly reduce patient spending on medications, even in the short term.

## Teaser

This cohort study analyzes out-of-pocket spending and dispensing of prescription drugs for mental health and substance use disorders after the implementation of a New Mexico law that prohibited cost-sharing for such treatments.

## Introduction

Underuse of mental health and substance use disorder (MH/SUD) treatment is a major public health challenge in the US,^1^ contributing to the high burden of MH/SUD-associated morbidity and mortality.^2,3,4,5^ People with MH/SUDs often delay or avoid seeking health care due to a range of factors including stigma and a lack of accessible clinicians.^6^ For commercially insured individuals, another common barrier is out-of-pocket expenses due to cost-sharing.^7,8,9^ Cost-sharing (including copayments, coinsurance, and deductibles) has increased over time^10,11^ and is intended to reduce overuse of health care. But for people with MH/SUDs, out-of-pocket cost can discourage use of effective treatments.

Cost-sharing discourages the use of all health care, a pattern most clearly illustrated by the RAND Health Insurance experiment^12^ in the 1970s, which randomly assigned participants to insurance plans with varying levels of cost-sharing. Reductions in mental health care in response to higher cost-sharing were larger than declines in other health care use.^12^ Since the RAND experiment, cost-sharing has become more pervasive in commercial insurance.^11^ However, there are also greater protections for people with MH/SUDs in the commercial market. For example, the landmark Mental Health Parity and Addiction Equity Act of 2008 (MHPAEA) required insurance plans covering MH/SUD treatment to do so in an equivalent manner to medical or surgical care. The few post-MHPAEA era studies^13,14^ suggest that cost-sharing continues to deter MH/SUD care, with mental health care use decreasing when people were switched from low-deductible to high-deductible insurance plans. Additionally, the MHPAEA has not been shown to substantially increase mental health care use.^13,14^

Policymakers have expressed interest in directly regulating cost-sharing in commercial insurance, but little is known about how state mandates around cost-sharing might influence patient outcomes. In April 2021, New Mexico passed SB 317, No Behavioral Health Cost Sharing (hereafter, NCS), into law. The NCS is a first-in-the-nation law prohibiting cost-sharing for MH/SUD treatment, including prescription medications, for people with commercial insurance plans and became effective January 1, 2022.^15^ The NCS was developed and passed based on a perceived need to reduce costs of MH/SUD treatment^16^ given the vast personal and societal costs of these conditions and the frequency with which cost is cited as a barrier to receiving treatment. As a state law, the NCS is only applicable to state-regulated commercial insurance plans, which include fully insured, employer-sponsored plans; individual market plans; and New Mexico state employee health plans (SEHPs), which include public school employees. The NCS does not affect self-insured employer plans, Medicare, Medicaid, or Indian Health Services.

This study examines the early outcomes of the NCS for dispensed psychotropic medications, focusing on people enrolled in New Mexico SEHPs compared with New Mexico residents enrolled in federal employee plans, which were not subject to the NCS. We hypothesized that the NCS would be associated with decreased out-of-pocket spending and increased dispensed medications among New Mexico SEHP enrollees compared with people unaffected by the law. However, the magnitude of these changes was uncertain because actions taken by insurers or pharmaceutical companies to reduce coverage generosity (eg, formulary or network restrictions) could mitigate the law’s impact. The impact could also be blunted by systemic factors such as low clinician availability or stigma.

## Methods

### Study Design and Participants

This retrospective cohort study was ruled exempt from review by the University of Minnesota institutional review board and did not require informed consent because the study used deidentified data in accordance with the Common Rule. This study followed the Strengthening the Reporting of Observational Studies in Epidemiology (STROBE) reporting guideline. The 2021 to 2022 IQVIA Longitudinal Prescription Claims Database was used, which includes approximately 90% of prescriptions filled in US retail pharmacies along with 60% to 85% of prescriptions filled via mail-order pharmacies, and provides detailed information on dispensed medications including drug name, class, and whether the drug is branded or generic; month dispensed; and information on the payer’s and prescriber’s state of residence.^17^ The prescription-level data were aggregated by drug class, month, and treatment or comparison group. One specific intervention group that was directly affected by NCS was identifiable in IQVIA data: New Mexico state employees. We could not accurately identify New Mexico patients with fully insured, employer-sponsored or individual market plans, and were unable to include such patients in the treatment group. We identified a comparison group similar to New Mexico state employees but whose insurance benefits were unaffected by NCS: federal employees who were dispensed medications prescribed by a clinician in New Mexico. In addition, monthly data on the number of state and federal employees in New Mexico were obtained from the Bureau of Labor Statistics.^18^

### Study Outcomes

The main outcomes were the mean nominal patient out-of-pocket spending for MH/SUDs and the monthly volume of dispensed medications primarily used to treat MH/SUDs. The data included information on dispensed medications including antidepressants, antipsychotics, anxiolytics, mood stabilizers, stimulants, opioid antagonists, medications for drug and alcohol dependence, and smoking deterrent medications. More detail on the included Uniform System of Classification level-5 drug classes is available in eTable 1 in Supplement 1. Mean out-of-pocket spending was expressed as spending per dispensed prescription, and monthly prescription volume was expressed as the number of dispensed prescriptions per 1000 employees, corresponding to the monthly measures of employees in the treatment and comparison groups. Secondary outcomes included mean out-of-pocket spending per prescription and prescription volume for drugs used primarily to treat MH disorders and drugs used to treat SUDs (eg, opioid use disorder, alcohol use disorder, and tobacco use disorder), branded or generic drugs, for drugs with above-median or below-median average out-of-pocket cost, and for new and existing MH/SUD prescription drug episodes. An episode was defined as any dispensed prescriptions within a drug class without a break of 60 days or more in medication supply. We categorized a prescription as new if the individuals had not had it dispensed or had been in possession of it for at least 60 days; otherwise, a prescription was categorized as existing (eg, an ongoing filled prescription). We also examined mean out-of-pocket spending and dispensed prescription volume for 3 other drug classes (asthma, cardiovascular, and diabetes) unaffected by the NCS but with a similar age profile as users of drugs for MH/SUDs.

###  Statistical Analysis

We conducted our study using a difference-in-differences estimation approach with observational data. Difference-in-differences analysis is a commonly used methodology for evaluating public policies,^19^ where trends in outcomes for a treatment group affected by a policy change are compared with trends for groups unaffected by the policy change.^19^ The key assumption in a difference-in-differences analysis is that the treatment and comparison groups would have followed the same trends in outcomes had the treatment (eg, the NCS law) not occurred. While this assumption is untestable because counterfactual outcomes for people enrolled in New Mexico SEHPs postpolicy are not observed, we followed the literature^20,21^ and examined trends in outcomes prior to passage of the NCS law. Details on the test of parallel trends in outcomes in the prepolicy period are included in the eAppendix in Supplement 1.

Linear regression models of the study outcomes were estimated with 3 independent variables: an indicator variable for whether an observation was observed after NCS implementation in January 2022, an indicator variable for whether an observation was in the treatment group or comparison group, and the interaction between being in the treatment group and being after the implementation of NCS. The key variable of interest was the interaction term, and the associated coefficient estimate represented the change in outcomes after NCS implementation for the treatment group. The regression model also included indicators for calendar month to adjust for seasonality. Regression models were unweighted and were estimated with robust standard errors to account for serial correlation in the outcomes. Analyses were conducted with Stata statistical software version 17.0 (SAS Institute).

Five secondary analyses examined the outcomes of NCS in greater detail. First, we examined MH and SUD drugs separately to test if patients responded similarly to NCS across the 2 drug classes. Second, we examined branded and generic drugs. Third, we separately examined new prescriptions compared with prescriptions continuing an existing episode. Fourth, we examined prescriptions for drugs that were above-median and below-median out-of-pocket cost. Fifth, we examined non–MH/SUD drugs unaffected by NCS. A 2-sided *P* < .05 was considered statistically significant. Data analysis occurred from December 2022 to January 2024.

## Results

**Figure 1.**
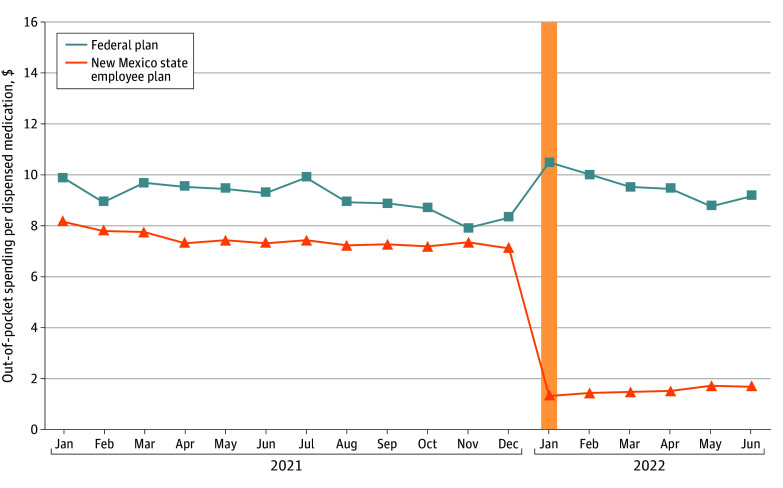
Mean Out-of-Pocket Spending per Dispensed Mental Health and Substance Use Disorder Medications The data source is the IQVIA Longitudinal Prescription Claims Database. Data were aggregated to the treatment group-month level. The *P* value for test of parallel preintervention trends is *P* = .20. The vertical yellow bar indicates the implementation of the No Behavioral Cost Sharing Law.

**Figure 2.**
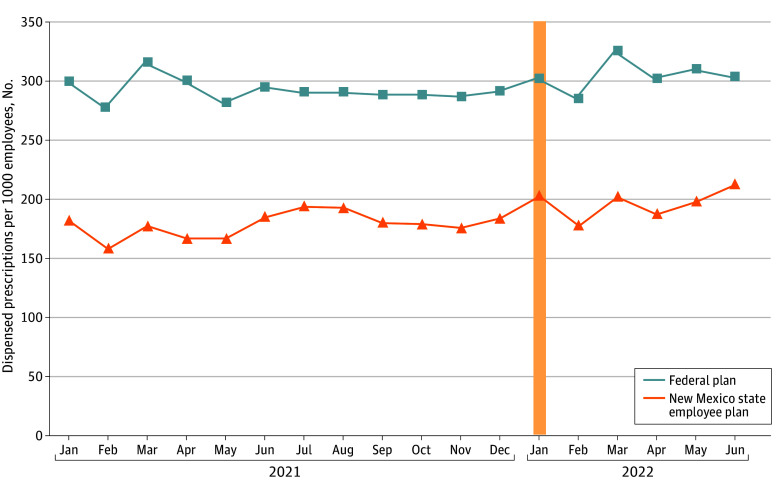
Mean Number of Dispensed Mental Health and Substance Use Disorder Prescriptions per 1000 Employees The data source is the IQVIA Longitudinal Prescription Claims Database. Data were aggregated to the treatment-group month level. The *P* value for test of parallel preintervention trends is *P* = .02. The vertical yellow bar indicates the implementation of the No Behavioral Cost Sharing Law.

The cohort included a total of 47 229 individuals (mean [SD] age 49.0 [19.6] years), including 21 626 individuals prescribed a total of 176 762 MH/SUD drugs in the treatment group and 25 669 individuals prescribed a total of 156 502 MH/SUD drugs in the comparison group. Table 1 shows the levels of the outcome variables for the full sample, the treatment and comparison groups, and different categories of drugs. Figure 1 shows the unadjusted monthly mean out-out-pocket spending per dispensed prescription for MH/SUD drugs for the treatment and comparison groups. People enrolled in New Mexico SEHPs exhibited a slight linear decline in out-of-pocket spending in 2021, which then dropped precipitously in January 2022. The trend for people enrolled in federal employee insurance plans in New Mexico followed a similar pattern in 2021, and then appeared to repeat the pattern for the first 6 months of 2022. There were no statistically significant differences in mean out-of-pocket spending per dispensed prescription across the treatment and comparison groups in the prepolicy change period. Figure 2 shows the unadjusted monthly trends in mean prescriptions dispensed per 1000 employees. The trends for the New Mexico SEHP group and for people enrolled in federal employee insurance plans in New Mexico were comparable throughout 2021, with the level of dispensed prescriptions slightly higher for the federal employee group. The test for parallel prepolicy change in number of dispensed prescriptions shows a small, statistically significant (coefficient estimate, 2.03; 95% CI 0.41-3.65; *P* for trend = .02) increase in the treatment group’s trend compared with the comparison group. The existing trends for both groups appeared to continue into the first 6 months of 2022.

**Table 1.** Summary Statistics^a^

Drug type	Overall sample (N = 47 229)			NM state employee health plan group (n = 25 669)^b^			NM federal employee group (n = 21 626)^c^		
	Claims, No. (%)	OOP spending per dispensed drug, mean (SD), $	No. of dispensed drugs per 1000 employees, mean (SD)	Claims, No. (%)	OOP spending per dispensed drug, mean (SD), $	No. of dispensed drugs per 1000 employees, mean (SD)	Claims, No. (%)	OOP spending per dispensed drug, mean (SD), $	No. of dispensed drugs per 1000 employees, mean (SD)
MH/SUD drugs									
Total	333 264 (100)	7.22 (1.52)	223.15 (12.42)	176 762 (53)	5.46 (2.88)	183.58 (14.00)	156 502 (47)	9.26 (0.63)	295.27 (12.27)
MH drugs	324 146 (97)	7.10 (1.48)	217.05 (11.95)	172 570 (52)	5.33 (2.82)	179.23 (13.51)	151 576 (45)	9.16 (0.62)	285.98 (11.63)
SUD drugs	9118 (3)	11.57 (2.98)	6.11 (0.64)	4192 (1)	11.03 (6.15)	4.35 (0.61)	4926 (2)	12.20 (1.43)	9.30 (0.91)
Generic	322 730 (97)	5.70 (1.26)	216.10 (13.26)	171 730 (52)	4.55 (2.36)	178.35 (15.03)	151 000 (45)	7.06 (0.31)	284.90 (12.71)
Branded	10 534 (3)	54.94 (8.12)	7.05 (1.87)	5032 (1)	35.86 (21.20)	5.23 (1.92)	5502 (2)	72.59 (18.98)	10.37 (1.98)
New drug episode	50 855 (15)	6.22 (1.11)	34.05 (1.99)	22 186 (7)	5.33 (2.25)	23.05 (2.18)	28 669 (9)	6.95 (0.67)	54.10 (2.79)
Existing drug episode	282 409 (85)	7.40 (1.60)	189.10 (10.79)	154 576 (46)	5.48 (2.99)	160.53 (12.06)	127 833 (38)	9.78 (0.73)	241.18 (10.76)
Non–MH/SUD drugs	1 584 932 (100)	12.83 (0.65)	260.85 (14.57)	869 207 (55)	10.49 (0.45)	215.36 (14.99)	715 725 (45)	15.50 (1.05)	343.72 (15.20)

Abbreviations: MH, mental health; NM, New Mexico; OOP, out of pocket; SUD, substance use disorder.

^a^ The data source is the IQVIA Longitudinal Prescription Claims Database.

^b^ Filled prescriptions in the state employee health plan group had mean (SD) patient age of 43.9 (15.2) years and 121 243 of 176 762 prescriptions (68.6%) were for women.

^c^ Filled prescriptions in the federal employee group had mean (SD) patient age of 52.9 (21.2) years and 109 223 of 156 502 (69.8%) were for women.

In the main regression models (Table 2), we found that compared with federal employees in New Mexico, there was a significant reduction in out-of-pocket spending after NCS went into effect. Mean (SE) out-of-pocket spending was reduced by $6.37 ($0.30) per prescription for New Mexico state employees compared with federal employees in New Mexico (95% CI, −$7.00 to −$5.75; *P* < .001). Compared with the pre-NCS sample, means for New Mexico state employees, this estimate corresponded to an 85.6% relative reduction in out-of-pocket spending per prescription. The difference-in-differences coefficient estimate for number of MH/SUD prescriptions dispensed per 1000 employees was not significant (5.79) when comparing New Mexico state employees with federal employees in New Mexico (95% CI, −6.14 to 17.71; *P* = .33). This nonsignificant estimate corresponded to a 3.3% relative increase compared with the pre-NCS mean for the treatment group.

**Table 2.** Difference-in-Differences Regression Results

Difference-in-differences model: NM state employees vs federal employees	Post–NCS, regression coefficient (SE)	*P* value	Pre–NCS treatment group, mean (SD)	Change from pre-NCS to post–NCS, %
Out-of-pocket spending per dispensed drug, $	−6.37 (0.30)	<.001	7.44 (0.31)	−85.6
Dispensed drugs per 1000 employees, No.	5.79 (5.73)	.33	177.47 (10.56)	3.3

Abbreviations: NCS, no behavioral health cost-sharing; NM, New Mexico.

The unit of analysis is the treatment and comparison group-month (36 months total). Regression models were adjusted for monthly seasonality. Percent change is relative to the pre-NCS treatment group mean.

Table 3 shows the secondary analyses results. The difference-in-differences coefficient estimates are shown in Table 3, along with the implied percent change compared with the pre-NCS outcome in the treatment group. NCS was associated with significant reductions in mean out-of-pocket spending that were similar in terms of relative magnitudes for dispensed MH and SUD medications. Volume of dispensed prescriptions for both MH and SUD medications did not change significantly when comparing the treatment group with federal employees in New Mexico. When stratifying by therapeutic class, we found significant reductions in mean out-of-pocket spending per prescription for all classes except for smoking deterrents (eTable 3 in Supplement 1). We did not find any changes in the number of dispensed prescriptions that were significant for any specific therapeutic class (eTable 3 in Supplement 1).

**Table 3.** Difference-in-Differences Regression Results by Prescription Drug Characteristic^a^

Drug Characteristic	Mean OOP spending per dispensed drug, coefficient estimate, $ (% change)	No. of dispensed drugs per 1000 employees, coefficient estimate (% change)
MH drugs	−6.24 (−85.9)^c^	5.87 (3.4)
SUD drugs	−12.14 (−80.7)^c^	−0.09 (−2.2)
Generic drugs	−5.03 (−81.6)^c^	4.65 (2.6)
Branded drugs	−71.60 (−148.1)^c^	1.14 (19.2)^b^
Low OOP-cost drugs	−4.03 (−74.1)^c^	3.23 (2.2)
High OOP-cost drugs	−18.61 (−90.6)^c^	2.56 (7.8)^b^
New drug episode	1.14 (−62.2)^c^	−0.04 (0.2)
Existing drug episode	−6.73 (−89.4)^c^	5.83 (3.8)
Non-MH/SUD drugs	1.69 (14.5)^c^	−6.84 (−3.4)

Abbreviations: MH, mental health; NCS, no behavioral health cost-sharing; OOP, out of pocket; SUD, substance use disorder.

^a^ The unit of analysis is the treatment and comparison group-month (36 months total). All models were adjusted for monthly seasonality. Each cell represents the estimated absolute change associated with NCS from the regression model, with the estimated change relative to the pre-NCS treatment group mean in parentheses.

^b^ *P* < .05.

^c^ *P* < .01.

NCS was associated with significant reductions in out-of-pocket spending for both generic and branded drugs (Table 3). However, the relative reduction in out-of-pocket spending per prescription was greater for branded drugs than generic drugs. There was no significant change in prescriptions dispensed for generic drugs, but there was a significant 19.2% relative increase (coefficient estimate, 1.14; 95% CI, 0.03-2.24; *P* < .04) for branded drugs. A similar pattern was found for more-expensive and less-expensive drugs. The relative reduction in out-of-pocket spending per prescription was greater for higher-cost drugs than lower-cost drugs, and there was a significant increase (Mean [SE] or coefficient estimate 2.56; 95% CI, 0.49-4.63; *P* = .02) in prescriptions dispensed for higher-cost drugs.

NCS was associated with significant reductions in out-of-pocket spending for both new and existing treatment episodes, though the relative magnitude was larger for existing episodes. However, NCS was not significantly associated with changes in the volume of dispensed medications for either new or existing treatment episodes. Finally, NCS was associated with a statistically significant but modest increase in mean out-of-pocket spending for non–MH/SUD drug classes. There was no association of NCS with volume of dispensed non–MH/SUD medications.

## Discussion

In this cohort study, we analyzed the New Mexico NCS law, which prohibits commercial insurance plans subject to state regulation from charging enrollees out-of-pocket payments for MH/SUD treatments. In examining trends in dispensed medications primarily used to treat MH/SUDs following the January 2022 implementation of the NCS among enrollees in the New Mexico SEHP, we found that mean out-of-pocket spending on psychotropic medications fell by $6.37, an 85.6% decrease, compared with federal government employees in New Mexico unaffected by NCS, suggesting that NCS effectively shielded patients from out-of-pocket spending on medications for MH/SUDs.

Medications are often a major financial burden for patients with MH/SUDs. In 2019, prescribed medications accounted for 29% of all US expenditures for treating mental health disorders,^22^ and average prices for branded drugs have increased much more rapidly than generic drugs in recent years, contributing more heavily to patients’ spending burden.^23^ Our analysis found relative out-of-pocket spending decreases were similar for medications primarily indicated for MH vs SUDs, suggesting cost-sharing reductions were widely felt among patients. The absolute and relative decrease in out-of-pocket spending was considerably larger for branded medications compared with generic medications. This finding suggests the law may have had a disproportionate impact on people using branded medications, which are often still under patent protection and have much higher average out-of-pocket costs than generic medications and can inform broader policy efforts to address the effects of newer medication costs on patients.

In contrast, the total volume of medications for MH/SUDs dispensed in the first 6 months after the NCS implementation for SEHP enrollees did not significantly change compared with residents with a federal employee plan. The only setting where we found NCS was associated with volume of drugs for MH/SUD was for branded drugs, where we also found the largest relative decreases in mean out-of-pocket spending per prescription. Most of prescriptions in our data are for generic drugs, but these results suggest the NCS law may be having intended early effects on access to branded drugs. We did not find an association of NCS with the volume of dispensed non–MH/SUD drugs, which were not targeted by the law. We did find a small increase in mean out-of-pocket spending for non–MH/SUD drugs associated with NCS, possibly suggesting the association of NCS with MH/SUD drug mean out-of-pocket spending might be understated, to the extent that there were increases in drug cost-sharing across all drugs.

Several factors in New Mexico may affect the lack of overall patient response to the NCS law. The study examined a brief period following the law’s implementation and prescription drug dispensing responses to the law could increase with time as both patients and clinicians become more aware of the law. The effects of NCS on MH/SUD medications could increase with time to the extent that more patients start seeking care for MH/SUDs. The elimination of cost-sharing may have had relatively smaller effects on the vast majority of dispensed medications that were generic and thus relatively inexpensive. Branded pharmaceutical companies could have responded to NCS by altering promotional activities or the use of coupons for affected medications, potentially offsetting some effects of the law change, and potentially explaining our null findings for medication volumes. Unfortunately, we cannot study such behaviors with our data, and this is an interesting direction for future research. The finding that the volume of psychotropic medications dispensed did not change may also reflect persistent nonmedication barriers in New Mexico such as low accessibility of prescribers and stigma that continued to limit MH/SUD treatment use despite the removal of cost barriers.

The findings of muted patient response are also consistent with prior studies showing MHPAEA did not substantially increase MH/SUD treatment use.^24,25^ Our results may also reflect increased high-deductible health plan enrollment, in which NCS only applies once patients meet their high deductible. Prior studies have shown that mental health care use declined after switching into such plans.^24,25^ Our study can be compared with the Patient Protection and Affordable Care Act Medicaid expansion which effectively made psychotropic medications available without cost-sharing. Similar to our results, a study^26^ found that the Medicaid expansion resulted in increased access to Medicaid-reimbursed prescription psychiatric medications but no change in total use, although other studies^27,28^ found larger increases in MH/SUD prescriptions after Medicaid expansion.

### Limitations

This study had several limitations. First, the data were limited to the first 6 months after the NCS law went into effect, and we are unable to assess the effect of NCS after that period. Second, we used aggregated data on dispensed prescription medications from specific treatment and comparison groups that could be reliably and consistently identified in the data. Therefore, we are unable to extrapolate our results to the entire affected population in New Mexico, or to compare with outcomes for other, nonpharmaceutical MH/SUD services (eg, counseling). We are also unable to separately examine the proportion of enrollees receiving any drugs and the volume of drugs conditional on any receipt. Third, the 2021 to 2022 study period happened during the COVID-19 pandemic, likely affecting trends in MH/SUD treatment. However, this limitation is unlikely to substantially affect the results because the intervention and comparison groups were similarly affected by the pandemic, and patterns of MH and SUD treatment had largely stabilized by 2021.^29^ Fourth, we found a small but statistically significant differential trend in the volume of dispensed MH/SUD prescriptions in the 12 months prior to the policy change, although this is unlikely to alter our conclusion that there was no overall association of NCS with the volume of dispensed MH/SUD prescriptions in the first 6 months after the law’s implementation. Fifth, we only observed dispensed prescriptions and have no information on prescriptions that were written but not dispensed or whether patients took dispensed medications. We also are unable to disentangle any associations of NCS with access to prescribing clinicians from the dispensing of prescribed medications for MH/SUDs.

## Conclusions

This cohort study makes important contributions to our understanding of the potential impact of prohibiting cost-sharing for MH/SUD treatment, finding that in the 6 months after New Mexico prohibited cost-sharing for MH/SUD treatment for state regulated commercial insurance plans, mean out-of-pocket spending for medications used to treat MH/SUDs declined. However, we also found no evidence of an association of NCS with the overall volume of dispensed MH/SUD prescription drugs in the first 6 months after the law was implemented. While our preliminary data suggest the law may have been successful in its goals of improving MH/SUD treatment affordability, the longer-term and broader effects of this law, such as on nonpharmaceutical services, remain to be seen. Nevertheless, the law may reduce the burden of psychotropic medication out-of-pocket expenses even in its first months of implementation, and potentially may help address the ongoing challenge of ensuring that financial barriers do not limit access to and quality of care for treatment of MH and SUDs.

## Notes

Supplement 1. **eTable 1.** List of Uniform System of Classification Level-5 Drug Classes Included in the IQVIA Data **eAppendix.** Statistical Test of Parallel Trends **eTable 2.** Results of Pre–NCS Parallel Trends Test **eTable 3.** Difference-in-Difference Regression Results by Prescription Drug Class

Supplement 2. Data Sharing Statement
