# Supplementary material for: Prescription Drug Dispensing and Patient Costs After Implementation of a No Behavioral Health Cost-Sharing Law
Source: JAMA Health Forum. 2024 Mar 22;5(3):e240198. doi: 10.1001/jamahealthforum.2024.0198 (PMC10960196; doi:10.1001/jamahealthforum.2024.0198)
Supplement: Supplement 1. — eTable 1. List of Uniform System of Classification Level-5 Drug Classes Included in the IQVIA Data eAppendix. Statistical Test of Parallel Trends eTable 2. Results of Pre–NCS Parallel Trends Test eTable 3. Difference-in-Difference Regression Results by Prescription Drug Class [file jamahealthforum-e240198-s001.pdf]

Supplemental Online Content

Golberstein E, Campbell JM, Maclean JC, Harris SJ, Saloner B, Stein BD. Prescription drug dispensing and patient costs after implementation of a no behavioral health cost-sharing law. *JAMA Health Forum.* Published online March 22, 2024. doi:10.1001/jamahealthforum.2024.0198

eTable 1. List of Uniform System of Classification Level-5 Drug Classes Included in the IQVIA Data

eAppendix. Statistical Test of Parallel Trends

eTable 2. Results of Pre–NCS Parallel Trends Test

eTable 3. Difference-in-Difference Regression Results by Prescription Drug Class

This supplemental material has been provided by the authors to give readers additional information about their work.

© 2024 Golberstein E et al. *JAMA Health Forum.*

eTable 1. List of Uniform System of Classification Level-5 Drug Classes Included in the IQVIA Data

PHENOTHIAZINE DERIVATIVES
ANTIPSYCHOTIC COMBINATION
ANTIPSYCHOTICS, OTHER
TRICYCLICS & TETRACYCLICS
MAO INHIBITORS
NEWER GENERATION ANTIDEPRESSANT
SSRI
SNRI
SSRI/5HT PARTIAL AGONIST
ANTIDEPRESSANTS IN COMBINATION
ANTIDEPRESSANTS, OTHER
ANTI-MANIA
ANALEPTICS/ CNS STIMULANTS
BENZODIAZEPINES
ANTIANXIETY, OTHER
NEWER GENERATION PSYCHOTHER AGENTS
PSYCHOTHERAPEUTICS, OTHER
SMOKING DETERRENTS
OPIOID REV AGENTS
DRUG DEPENDENCE

© 2024 Golberstein E et al. *JAMA Health Forum.*

eAppendix. Statistical Test of Parallel Trends.

To assess whether the trends in the outcomes were trending similarly in the period before the implementation of NCS, we estimated regression models of both of the study outcomes. The regression models were restricted to the 24 observations in the pre-policy change period (2021). The regression models included an indicator for being in the treatment group, a set of indicators for each of the 12 months, and the interaction between being in the treatment group and a linear measure of time measured in months. The coefficient on the interaction term measures the degree to which the treatment group was trending differently than the comparison group in the period prior to the policy change. The results of these models are in Table A1.

eTable 2. Results of Pre-NCS Parallel Trends Test

	(1)<br>Average OOP$ per Dispensed Rx	(2)<br>Dispensed Rx per 1000 Employees
NCS*Month	0.06	2.03
(SE)	(0.04)	(0.23)
P-value	0.203	0.019
Pre-NCS treatment group mean	$7.44	177.47

Note: N=24 for all regression models, the unit of analysis is the treatment/comparison group-month. Regression models adjust for monthly seasonality. Robust standard errors are reported in parentheses.

© 2024 Golberstein E et al. *JAMA Health Forum.*

eTable 3. Difference-in-Difference Regression Results by Prescription Drug Class

eTable 3 shows the results of the difference-in-differences analyses for specific drug classes.

	(1)	(2)
	Average OOP$ per Dispensed Rx	Dispensed Rx per 1000 Employees
A. Antidepressants	-$6.27***	4.95
	-91%	4%
B. Antipsychotics	-$12.41***	0.45
	-79%	5%
C. Anxiolytics	-$1.06***	-0.65
	-23%	-2%
D. Mood Stabilizers	-$4.63***	0.04
	-75%	4%
E. Stimulants	-$10.40***	1.19
	-67%	7%
F. Specific Antagonists	-$15.39***	0.09
	80%	3%
G. Smoking Deterrents	$9.03	-0.18*
	60%	-22%

Note: N=36 for all regression models, the unit of analysis is the treatment/comparison group-month. All models adjust for monthly seasonality. Each cell represents the estimated absolute change associated with NCS from the regression model, with the estimated change relative to the pre-NCS treatment group mean in brackets below. *p<.10, **p<0.05, ***p<0.01

© 2024 Golberstein E et al. *JAMA Health Forum.*
