# Supplementary material for: Prescription Drug Dispensing and Patient Costs After Implementation of a No Behavioral Health Cost-Sharing Law
Source: JAMA Health Forum. 2024 Mar 22;5(3):e240198. doi: 10.1001/jamahealthforum.2024.0198 (PMC10960196; doi:10.1001/jamahealthforum.2024.0198)
Supplement: Supplement 2. — Data Sharing Statement [file jamahealthforum-e240198-s002.pdf]

Data Sharing Statement

Golberstein. Prescription Drug Dispensing and Patient Costs After Implementation of a No Behavioral Health Cost-Sharing Law. *JAMA Health Forum*. Published March 22, 2024. doi:10.1001/jamahealthforum.2024.0198

Data
Data available: No

Additional Information
Explanation for why data not available: We use IQVIA data, and we are precluded from sharing the data per our Data Use Agreement.
